# Post-cholecystectomy Incisional Hernia With Gastric Outlet Obstruction

**DOI:** 10.7759/cureus.86169

**Published:** 2025-06-16

**Authors:** Saad Salman, Muhammad Haseeb Shah, Sofia Jehanzeb, Bashir Khan, Aurangzeb Khan

**Affiliations:** 1 Department of General Surgery, Mardan Medical Complex, Mardan, PAK

**Keywords:** abdomen ventral hernia, cholecystectomy, gastric hernia, gastric outlet obstruction, incisional hernia repair

## Abstract

An incisional hernia is a common complication after abdominal surgery. The omentum and small bowel are the most commonly herniated intra-abdominal structures through the fascial defect; however, involvement of the stomach in incisional hernia causing gastric outlet obstruction is very rare. We report a case of a 50-year-old female patient who presented with symptoms of intermittent nausea, vomiting, abdominal swelling, and postprandial discomfort. Clinical examination and imaging revealed an incisional hernia containing an antral part and greater curvature of the stomach, causing gastric outlet obstruction. Reduction of the herniated contents, defect closure, and on-lay mesh placement resulted in the resolution of symptoms. Gastric outlet obstruction secondary to incisional hernia is more frequently observed in middle-aged elderly patients who have had previous abdominal procedures and in women with multiple pregnancies. Contrast-enhanced computed tomography (CECT) plays a crucial role in the diagnosis. Surgical repair remains the definitive treatment for hernias to prevent complications. This case highlights the need to consider incisional hernias with gastric herniation as a potential cause for gastric outlet obstruction, especially in patients of geriatric age. Early diagnosis and surgical treatment are important to relieve symptoms and reduce the risk of complications.

## Introduction

An incisional hernia is a type of ventral hernia that occurs at the site of a previous surgical incision in the abdominal wall [1]. Longitudinal/vertical abdominal incisions have a higher risk of hernia compared to transverse or oblique incisions, with upper abdominal incisions having a higher risk of hernia than those in the lower abdomen [2].

In the literature, the incidence of incisional hernias ranges from 3.8% to 11.5% [3]. There are various risk factors identified for the development of incisional hernias, most commonly old age, obesity, smoking status, diabetes mellitus, renal failure, and the presence of surgical site infections [4].

In Pakistan, there is limited data available on the occurrence of incisional hernias following surgery. However, a study conducted in 2015 in Narowal, Pakistan, reported an incidence of 7.27% for incisional hernias [5].

Hernias typically contain a portion of the omentum or small intestine, as these are the most mobile intra-abdominal structures [6]. Incisional hernias causing gastric outlet obstruction are exceptionally rare, with only a few cases documented in the medical literature [7]. Gastric outlet obstruction is most commonly caused by malignancies or peptic ulcer disease [8]. Contrast-enhanced computed tomography (CECT) is useful in the diagnosis of gastric outlet obstruction [9].

Although acute management is conservative treatment with nasogastric (NG) tube decompression, definite treatment has always been the surgical reduction of intra-abdominal contents and hernia repair [10]. The rarity of incisional hernias leading to gastric outlet obstruction often results in these cases being overlooked in differential diagnoses, delaying diagnosis and treatment [11]. This report presents a rare case of gastric outlet obstruction caused by a post-cholecystectomy incisional hernia in an elderly female patient.

## Case presentation

**Figure 1:**
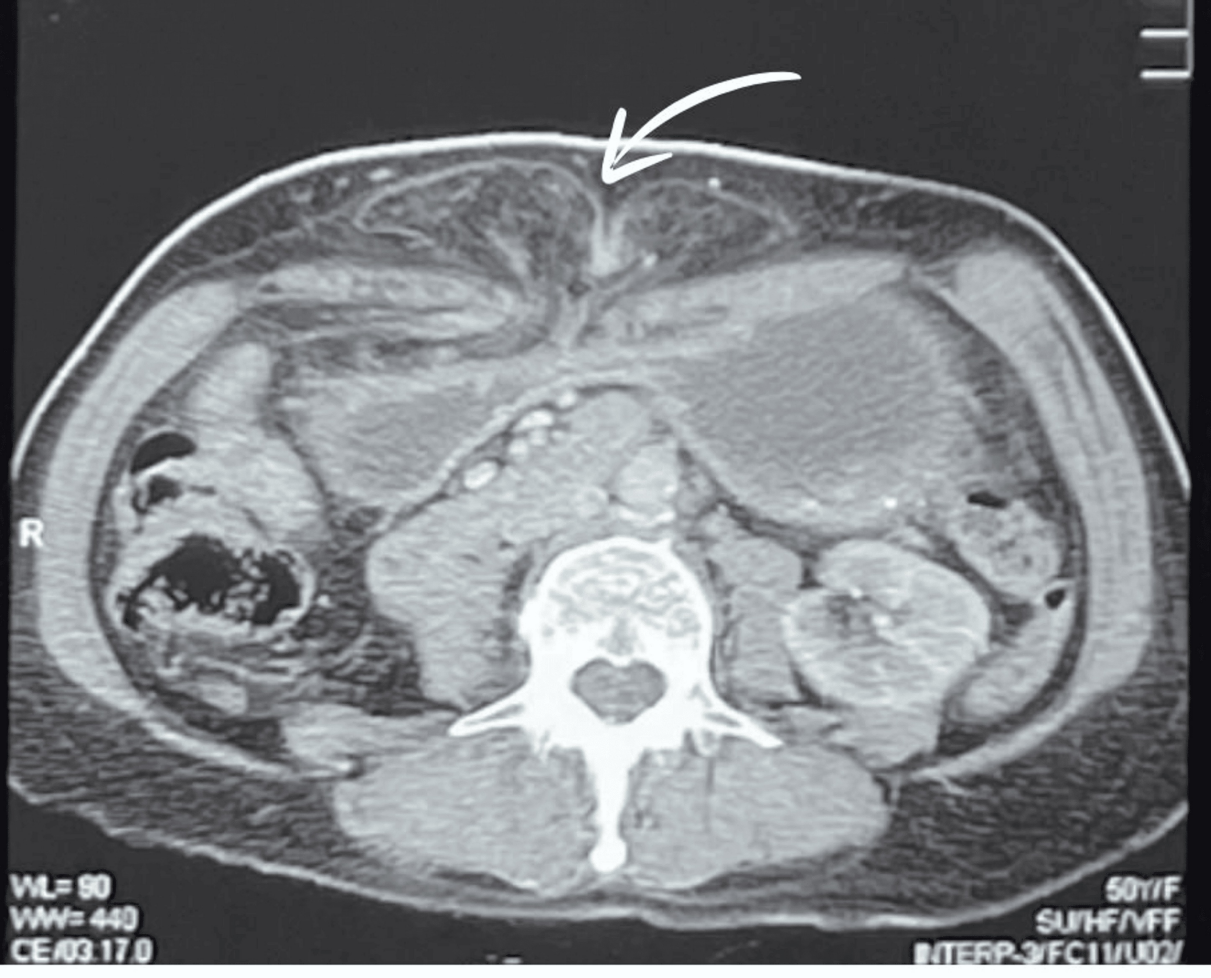
Axial view of the incisional hernia, stomach, and omentum can be seen protruding from the site of the previous incision

On January 22, 2025, a 50-year-old female patient presented to the surgical outpatient department, with the complaint of intermittent nausea and vomiting, accompanied by a swelling in the epigastric region. She had been experiencing these symptoms for the past two years, unresponsive to medical therapy. The patient had a medical history of hypertension, managed with amlodipine/valsartan 5/80 mg, a thyroidectomy with ongoing thyroxine 50 mg BD, and a previous open cholecystectomy two years back. She had multiple consultations with physicians and gastroenterologists for intermittent vomiting. A neurological consultation was also conducted, but no central cause was identified. Abdominal and pelvic ultrasound (USG) showed a 37 mm defect in the anterior abdominal wall at the epigastric region, with protrusion of abdominal contents and a positive cough impulse. A CECT scan was advised, which was performed on January 2, 2025, which reported gastric pylorus thickening, suspecting a neoplastic lesion, and recommended histopathological correlation. An upper gastrointestinal (GI) endoscopy was performed and revealed a grossly normal-appearing pylorus. However, intubation of the pylorus was unsuccessful due to anatomical distortion caused by gastric herniation through the incisional hernia, which had led to angulation and elongation of the prepyloric segment, resulting in misalignment of the pyloric channel. Suspecting the authenticity of the endoscopy report, a repeat CECT of the abdomen and pelvis was done on January 6, 2025, which demonstrated a markedly distended stomach filled with fluid, mild circumferential wall thickening of the pyloric antrum, and a large supraumbilical hernia with a 33 mm defect containing omental fat as seen in Figure 1.

The patient was referred by the gastroenterologist to the surgical outpatient department upon suspicion of gastric outlet obstruction secondary to herniation of the gastric antrum. Vital signs were stable (blood pressure (BP): 130/90 mmHg; heart rate (HR): 62 bpm; SpO_2_: 98%, afebrile). Physical examination was done in sitting and standing positions, which revealed a swelling in the epigastric region, along the medial margin of the previous right subcostal incision, suggestive of an incisional hernia. The patient’s symptoms were exaggerated on sitting and coughing and relieved upon lying down. The lab results are shown in Table 1.

**Table 1:** Lab results TSH: thyroid-stimulating hormone

Test	Result	Unit	Reference range
Leukocyte count	9,000	/μL	4,000-11,000/μL
Hemoglobin	13.7	g/dL	14-17 g/dL
Platelets	346,000	/μL	150,000-450,000/μL
Total bilirubin	0.8	mg/dL	0.1-1.2 mg/dL
Urea	39	mg/dL	15-40 mg/dL
Creatinine	1.31	mg/dL	0.7-1.25 mg/dL
TSH	2.92	μIU/mL	0.5-5.0 μIU/mL
Na	133	mmol/L	135-145 mmol/L
K	3.4	mmol/L	3.5-4.5 mmol/L
Cl	98	mmol/L	98-106 mmol/L

**Figure 2:**
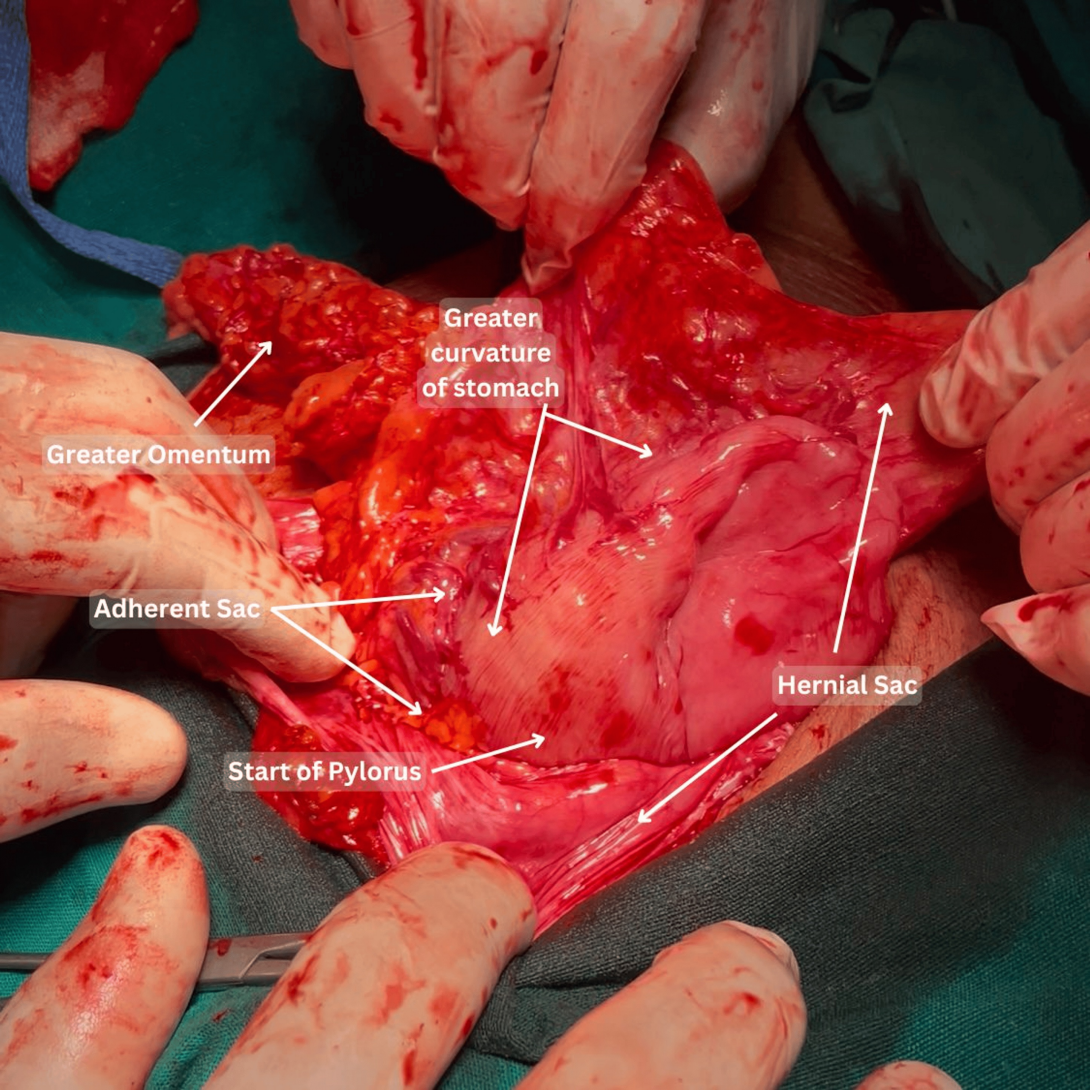
Intraoperative view of the incisional hernia with gastric involvement

**Figure 3:**
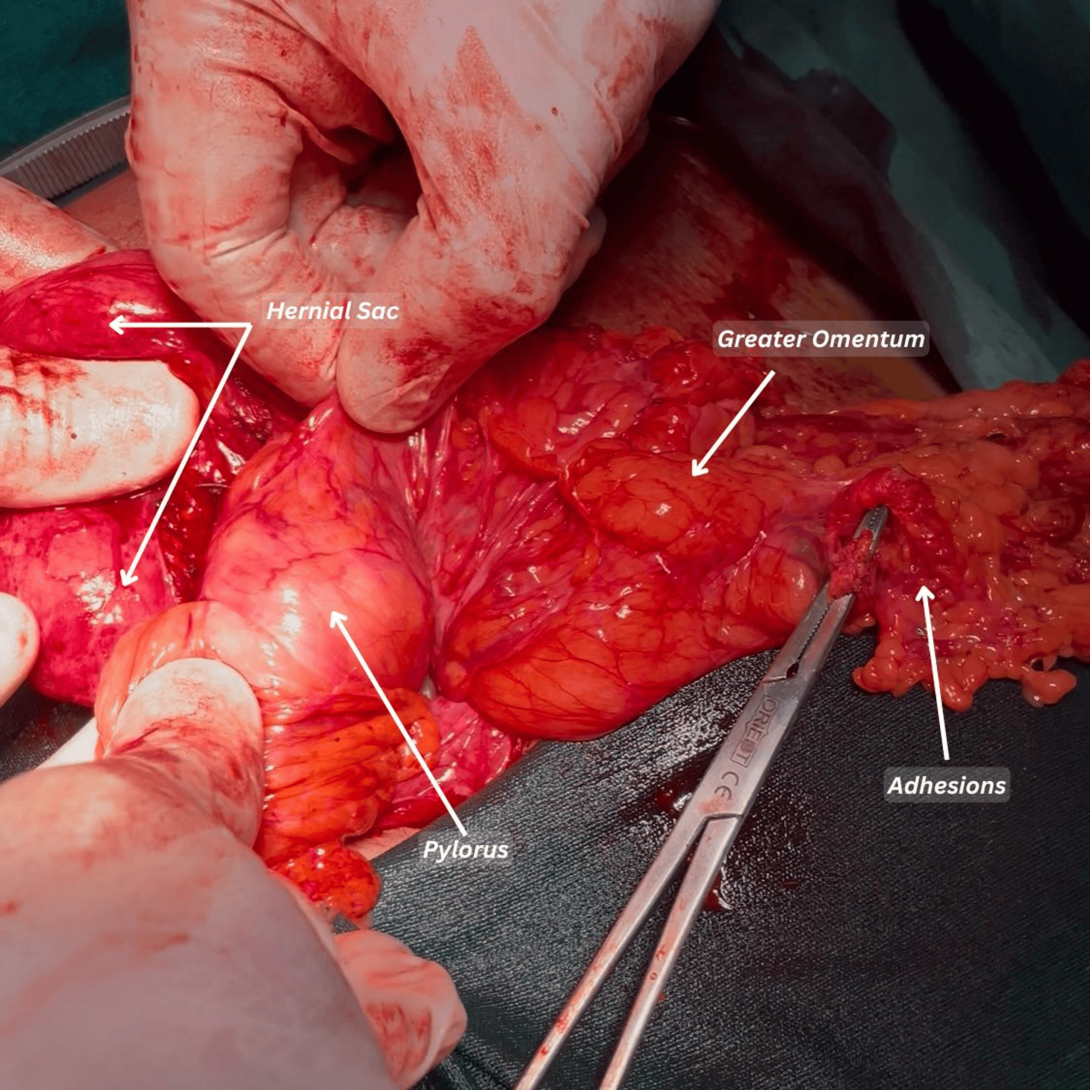
The image shows the pyloric end of the stomach adherent to the hernial sac

The mildly elevated creatinine was likely due to dehydration caused by persistent vomiting. The patient was initially managed with intravenous fluids, and informed consent was obtained. Following the completion of baseline investigations and preoperative optimization, the patient was scheduled for mesh repair. On February 14, 2025, surgical exploration via an upper midline incision was performed, which revealed herniation of the greater curvature and pyloric part of the stomach, along with omentum and transverse colon, through the abdominal wall defect. The hernial sac was found to be adherent to the greater curvature of the stomach (Figures 2, 3).

The herniated contents, including the stomach, omentum, and transverse colon, were reduced, and dense adhesions-particularly between the hernial sac and the greater curvature of the stomach-were carefully released. The abdominal wall defect was closed primarily, and a 15 × 15 cm polypropylene mesh was placed in an on-lay position, secured using interrupted non-absorbable polypropylene sutures. A closed suction subcutaneous drain was placed and removed on the second postoperative day. The patient received perioperative intravenous antibiotics and was advised to use an abdominal binder, avoid heavy lifting, and maintain light activity for six weeks to prevent recurrence or gastric re-herniation. Postoperatively, her symptoms resolved, and she was discharged on the second day without any complications. She has remained symptom-free during the ongoing follow-up period, with no signs of recurrence noted at one month postoperatively. Continuous surveillance is ongoing, and no complications or signs of recurrence have been observed to date.

## Discussion

Gastric outlet obstruction due to incisional hernia is a rare occurrence, attributed to the stomach's relatively fixed position in the abdomen due to its ligamentous attachments [12]. The position of the stomach is supported by several peritoneal ligaments that anchor it within the abdominal cavity [13].

The gastrohepatic ligament connects the lesser curvature of the stomach to the liver, providing superior support. The gastrosplenic ligament attaches the greater curvature to the spleen, containing the short gastric and left gastroepiploic vessels. The gastrocolic ligament, also called the supracolic omentum, extends from the greater curvature of the stomach to the superior and anterior surfaces of the transverse colon [14]. The gastrophrenic ligament links the fundus of the stomach to the diaphragm, offering superior stabilization [15]. These ligaments collectively ensure the stomach remains relatively fixed in its place, compared to the more mobile intestines [16].

Similar cases have been rarely reported in the literature. One such report described a 68-year-old woman with gastric outlet obstruction associated with a ventral hernia [17]. Another involved an 81-year-old with incarcerated pylorus in an umbilical port site hernia [8]. Additionally, a 64-year-old woman was reported to have an irreducible epigastric hernia containing the gastric antrum, also resulting in gastric outlet obstruction [18].

In all these cases, the patients were middle-aged or elderly women with gastric herniation leading to gastric outlet obstruction, sometimes resulting in severe dehydration and electrolyte imbalance. All these cases mentioned above were surgically treated; however, one case of gastric outlet obstruction secondary to an incarcerated inguinal hernia containing gastric antrum was successfully treated with percutaneous endoscopic gastrostomy (PEG) tube placement [10], as the patient had no signs of bowel ischemia or gastric perforation and they did not give consent for elective surgical repair. The position of the stomach can become lax in multiparous women, significantly increasing the risk of gastric herniation [12]. Aging and hormonal changes can weaken peritoneal ligaments such as the gastrohepatic, gastrosplenic, gastrocolic, and gastrophrenic, reducing stomach support and increasing mobility [16].

Additionally, weakening of the abdominal wall and diaphragm over time or after pregnancy decreases intra-abdominal support, making herniation more likely. Chronic intra-abdominal pressure from obesity, chronic coughing, or straining further contributes to herniation [1].

Although CECT is the imaging modality of choice for diagnosing incisional hernias with gastric outlet obstruction [19], in our case, the initial CT did not reveal the herniation, possibly due to patient positioning, transient nature of the hernia, or a diagnostic focus on suspected pyloric thickening, which raised concern for neoplasia. A subsequent CT, performed during a more symptomatic phase and with greater clinical suspicion, clearly identified the herniation and its contents. This underscores the importance of considering mechanical causes in persistent upper GI symptoms and the value of repeat or targeted imaging when initial studies are inconclusive. Dynamic USG of the abdominal wall has also proven to be an accurate alternative to CT for the detection of abdominal hernias, with a sensitivity and specificity of 98% and 88%, respectively [20].

## Conclusions

Gastric outlet obstruction due to an incisional hernia is a rare but important differential diagnosis, particularly in elderly patients with multiple comorbidities and a history of abdominal surgery. This case highlights how gastric herniation, likely facilitated by age-related or post-surgical weakening of peritoneal ligaments, can present with non-specific GI symptoms and be easily overlooked. In our case, CECT imaging was instrumental in identifying the herniated stomach and narrowing at the pylorus-findings that were not fully appreciated through endoscopy due to altered anatomical positioning. Surgical exploration confirmed the diagnosis, and reduction of the herniated contents with on-lay mesh repair led to complete symptom resolution. This emphasizes the importance of maintaining a high index of suspicion, using appropriate imaging when endoscopic findings are inconclusive, and proceeding to timely surgical intervention to prevent complications. Given the rarity of such case-especially in the Pakistani population-further research and documentation are needed to improve early recognition and guide optimal management strategies.
